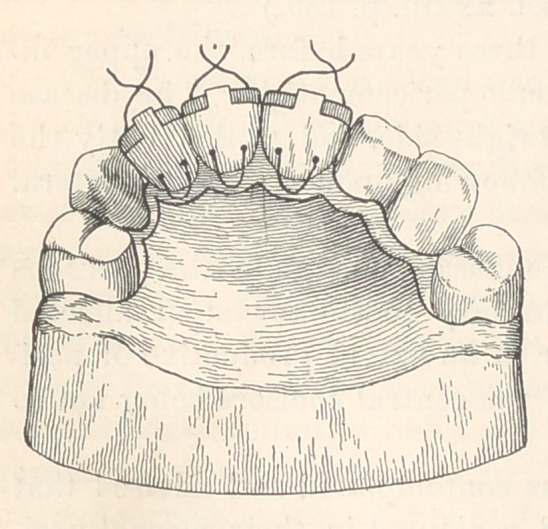# Academy of Stomatology

**Published:** 1897-10

**Authors:** 

**Affiliations:** Academy of Stomatology


### ACADEMY OF STOMATOLOGY.

A REGULAR meeting of the Academy of Stomatology was held in the rooms of the society, 1731 Chestnut Street, on April 27.

The proposed amendment to the constitution—viz., that the council of the society shall consist of six instead of five members, as heretofore—was voted upon and passed.

Dr. Thomas called attention to a deficiency of the subscription-list for the placing of an heroic bust of Dr. Horace Wells, the discoverer of anæsthesia, in Washington, D. C. Although the appeals to the profession at large for subscriptions had met with a prompt and generous response, the sum total is still insufficient to provide the proposed memorial. Other dental societies, as well as the dental fraternity in general, had subscribed, and Dr. Thomas urged that the members of the Academy subscribe again to so worthy an object as perpetuating the memory of one of the world's greatest benefactors.

The Clinic Committee reported an exhibition of a case by Dr. Louis Jack, upon whom he had performed two operations in the same mouth, which might be fittingly termed the reimplantation of teeth.

*Dr. Jack.*—The case herewith presented is one in which correction of a deformity and cure of a diseased condition have been accomplished by means of radical surgical measures. The case is described in my paper upon "Plantation of Teeth," *Proceedings of the Academy of Stomatology*, December, 1895, as number two of the replantation cases. (See INTERNATIONAL DENTAL JOURNAL, vol. xvii. p. 139; also *Dental Cosmos*, vol. xxxiii. p. 189.)

The patient, a female. About three years before, the upper incisor teeth fell victims to phagedenic pericementitis. The disease began in the lateral incisor of the right side, and subsequently the central incisors became involved; following upon this the left lateral incisor has become displaced.

The early clinical stages of the disease change in the positions of teeth; what is known as voluntary tooth-movement affected markedly the right lateral incisor to an extent productive of positive deformity, the malposition of the central incisors being not so marked.

As a means of correction it was contemplated and advised that the affected teeth be removed and replanted in their normal positions. To this the patient demurred, but consented that the operation be performed upon the tooth most out of alignment,—viz., the lateral incisor. As there was evidence of disappearance of a portion of the partition of bony tissue between this incisor and the adjoining central incisor, it was urged that the operation be performed upon both teeth, to which the patient would not assent.

She was etherized, the lateral incisor was extracted, its canal cleansed and filled with gutta-percha, and placed in an antiseptic solution. The existing abnormal alveolus was extended at its lingual aspect, and deepened, until by measurement it was shown that it would permit the placement of the tooth in correct position, —that is, virtually, although the tooth was replanted, a new socket was formed, so that the operation actually resembles implantation. The sterilized tooth was placed in position and retained immovably by means of a fixture for a period of six months, when the tooth was found to be perfectly firm in its new socket, and upon percussion exhibited normal resonance.

The malposition of the central incisors became so marked that operation upon them was deemed imperative. By this time the pocket upon the distal wall of the root of the right central incisor was extensive, exhibiting a loss of at least a portion of the adjoining wedge of alveolar process. The roots of these teeth were immersed in a dilute solution of hydrochloric acid (after the method of Dr. Amoedo), but not carried to the extent of decalcification advised by him, the purpose being to remove any minute deposits of calculi which had escaped detection, as well as to soften the cementum which had become exposed by the extension of the teeth.

The retaining appliance (see illustration) consisted of a narrow gold plate clasped to the bicuspid teeth ; a tongue of the plate extended over the lingual face of the replanted tooth, through which two holes were drilled near the neck portion ; an extension of the tongue, as shown, was carried over the cutting-edge of the tooth. Through the openings a fine platinum ligature was passed, and the teeth were lashed to the plate to maintain their position laterally. The illustration shows the fixture adapted to the retention of three teeth.

The splint for the fixation of the lateral was kept on for six months, but in the case of the centrals, at the end of four months the teeth were found firm in their sockets. Upon percussion the teeth exhibit normal resonance, except the right central incisor, that in which the degenerative changes accompanying phagedenic pericementitis were marked; this tooth responds with a dull, flat note when tapped, indicating an inferior density of the surrounding tissue in comparison with the two other replanted ones.

The gum festoons about the necks of the teeth exhibit a normal appearance, there being no evidence that the teeth have undergone operation. In addition, the crowns of the teeth are translucent, appearing as though they contained vital pulps.

The remaining lateral incisor is now involved, which will necessitate, in the near future, operation upon that tooth also. This operation is of interest as exhibiting the possibility of radical relief in the early stages of phagedenic pericementitis before atrophy of the alveolar walls occurs.

DISCUSSION.

*Dr. Guilford.*—I examined the case, and think it a pronounced success; the teeth are firm, and there is no evidence of operation upon the crowns or the roots of the teeth.

*Dr. Roberts.*—What is your opinion, Dr. Jack, of the mode of union in these cases?

*Dr. Jack.*—Histological data are deficient in this connection, but, combining such data as we have with reasoning upon pathological and physiological teachings, it appears to approach in nature an encystment, having the characteristics of ankylosis.

Dr. James Truman arose to a personal explanation. At a former meeting his remarks on Dr. Williams's slides were misinterpreted, and were made to appear as in opposition to Professor Miller's views on dental caries. Such interpretation was not warranted by anything then stated, and was widely at variance from his own views. His criticism, at the meeting mentioned, was confined entirely to two or three slides in the collection. The preparation of these, as well as all exhibited, was beyond criticism, but the deductions sought to be drawn from them were not, in his judgment, correct. He then proceeded to give his ideas in a manner to avoid misconception.

*The President.*—The papers of the evening are now in order,—the essay of John Girdwood, D.D.S., L.D.S., Edinburgh, Scotland, upon "Root-Perforation: A New Method of Treatment," and a short report by Dr. Henry C. Register upon "Some Features in Bridge-Work."

(For Dr. Girdwood's paper, see page 357, and for Dr. Register's paper, see page 641.)

*The President.*—These papers are now open for discussion.

*Question.*—Why should paraffin not be used for this purpose? Certainly it is, theoretically, an applicable material.

*Dr. Inglis.*—I have used paraffin for perforation near the apex of the root. It has the virtue of easy removal.

One difficulty of treating these cases of perforation near the apex is the sterilization and filling of that portion of the canal which lies beyond. There is always a doubt as to the condition of this apical portion of the canal. The first thing to do with a perforation is to diagnose it. The cases that I have seen have occurred in consequence of the lateral cutting of reamers which were not held in the axis of the tooth. A fine probe passed towards the apex causes pain and immediate hemorrhage. I have pursued the following method: A gutta-percha cone is introduced, and its point cut off, until no sensation is produced, when it is placed in the canal. This is coated with thick chloro-percha, and slowly but fully introduced; however, it had better be too short than too long.

For the large lateral perforations I use a plaque of gutta-percha. In one case of perforation with an external fistula overlying it, I pressed back the soft tissue, exposing the perforation through the gum; then placing a gutta-percha cone in the canal, I treated the perforation as a cavity,—filled it from the exterior with facing amalgam. The wound healed, and the tooth is now doing good service.

*Dr. Darby.*—My son had a case closely analogous in its relations to perforation of the root. A boy, aged eleven years, fell and drove his central incisors out of position. The teeth were replaced in position and held firmly until healing occurred. The pulps of the teeth which had been devitalized by the accident were removed, and it was found that the foramina were of immature size. I suggested that the canals be reamed to the size of the foramen; then, by measuring the exact distance from the floor of the pulp-chamber to the apex of the root, a gold wire was placed in the latter, and a shoulder made by cutting away the wire to the diameter of the reamer employed; the thinned portion was then cut off and the end rounded until it exactly equalled in length the length of the canal, the shoulder engaging and resting upon the floor of the pulp-chamber. The wire, dipped in thin chloro-percha, was placed in position and served as the canal filling.

I want to sound a warning as to the use of copper amalgam.

Placed in the crowns of pulpless teeth, it will certainly discolor the crowns if no impermeable layer be interposed between the filling and the dentinal walls.

*Dr. Register.*—It does not discolor roots in which it is placed.

*Dr. Darby.*—It will be discolored whenever placed in such situations as the decomposition of albuminous matter is found; wherever hydrogen sulphide is formed copper amalgam will discolor, and it very frequently is formed in pulpless teeth by the decomposition of the organic contents of the dentinal tubuli.

*Dr. Truman.*—There is one phase of this matter which is not receiving the attention it should in our discussion, and that is the condition of the parts as regards sepsis. The older the perforation the greater is the difficulty of producing sterilization, and until perfect antisepsis is attained, we must consider the danger of degeneration of the vascular tissues about the parts. The difficulty of successfully treating lateral perforations is greatly increased if the parts are permitted to become or remain in a septic condition.

*Dr. Register.*—The best treatment for perforation is the preventive treatment. If in reaming canals we perform the operation very carefully, it will be found that, as the instrument approaches or touches the cementum, the patient will give evidence of pain; if the patient be previously directed to state when sensation occurs, perforations should never occur. When I find that the instrument (the reamer) has invaded the cementum, I sterilize the canal thoroughly, and am careful to exercise no pressure in placing the root-filling.

Those cases of perforation near the apex which I have encountered I have treated after one method. Packing in the canal, and against the pericementum at the perforation, a small quantity of salol, and over this a cone of zinc phosphate is placed. Of course, the salol disappears, as it always does, after a period when used as a canal filling; but I believe that it performs office as an unirritating antiseptic while it lasts.

*Dr. Guilford.*—Accidents of perforation are always to be well guarded against. In those cases involving the floor of the pulp-chamber of molars, I cover them with a piece of moistened court-plaster and fill over it.

When the perforation is near the root-apex, I think it best to amputate the portion of root above the perforation. The canal is filled with gutta-percha, an opening is made through the alveolar wall above the point of perforation, and the apex of the root is cut off, and smoothed by means of fissure burs. The piece is dislodged by means of an elevator, and the wound cavity packed with iodoform gauze.

*Dr. Roberts.*—I have used tin-foil instead of court-plaster for this purpose, after sterilizing the parts. Stiff zinc phosphate is then to be packed over the tin-foil.

Adjourned.

HENRY H. BURCHARD,
*Editor Academy of Stomatology.*

## Figures and Tables

**Figure f1:**